# Supplementary material for: Conditional chemoconnectomics (cCCTomics) as a strategy for efficient and conditional targeting of chemical transmission
Source: eLife. 2024 Apr 30;12:RP91927. doi: 10.7554/eLife.91927 (PMC11060718; doi:10.7554/eLife.91927)
Supplement: Figure 3—source data 3. [file elife-91927-fig3-data3.zip › Figure 3 source data3 part 2.zip!/Figure 3-Source data 3-part2/1053f3-1-CRISPResso_Report_t6djfp6u.zip!/CRISPResso_on_1053f3_3/1c.Alignment_barplot.pdf]

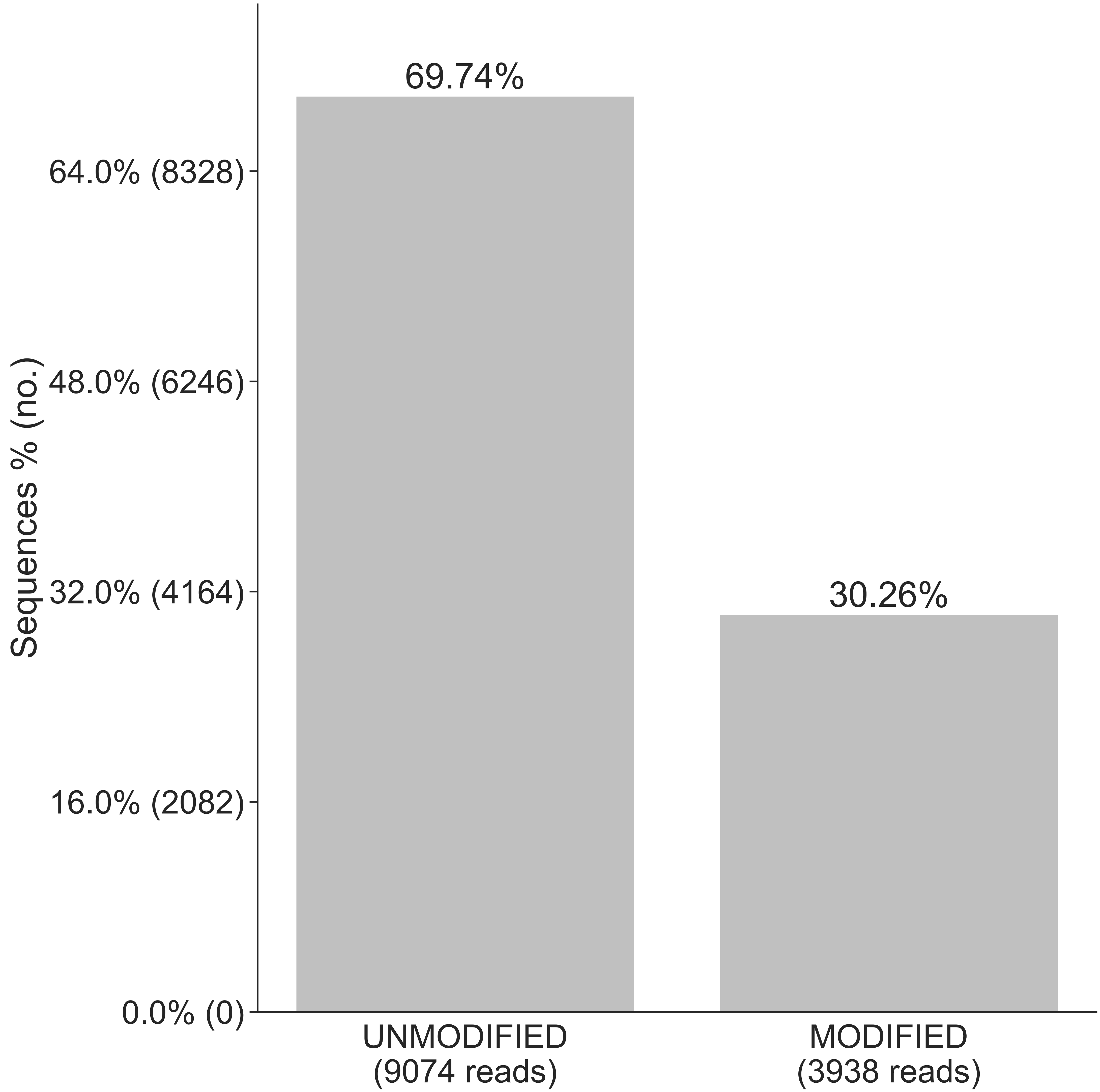
69.74%
30.26%
Sequences % (no.)
64.0% (8328)
48.0% (6246)
32.0% (4164)
16.0% (2082)
0.0% (0)
UNMODIFIED
(9074 reads)
MODIFIED
(3938 reads)
